# A Prenatal Presentation of CDK13-Related Disorder with a Novel Pathogenic Variant

**DOI:** 10.1155/2023/3437706

**Published:** 2023-06-14

**Authors:** Michael Gibbs, Alysa Poulin, Yanwei Xi, Bita Hashemi

**Affiliations:** ^1^Department of Pediatrics, Division of Medical Genetics, University of Saskatchewan, Saskatoon, Canada; ^2^Department of Pathology and Laboratory Medicine, University of Saskatchewan, Saskatoon, Canada; ^3^Department of Genomic Laboratory Pathology and Laboratory Medicine, University of Saskatchewan, Saskatoon, Canada

## Abstract

Cyclin-dependent kinase 13 (*CDK13*) is a member of the cyclin-dependent serine/threonine protein kinase family. Members of this family are well known for their essential roles as master switches in cell cycle control. CDK13-related disorder is a newly described genetic condition with characteristic clinical features including mild to severe intellectual disability, developmental delay, neonatal hypotonia, a variety of facial dysmorphism, behavioral problems, congenital heart defects, and structural brain abnormalities. We report a case of prenatal diagnosis of CDK13-related disorder. Detection of cystic hygroma with thickened nuchal fold led to prenatal genetic investigation, which identified a novel *de novo* likely pathogenic variant in the *CDK13* gene (c.900C > G, p.Tyr300^*∗*^). Pregnancy was terminated and autopsy was performed. To our best knowledge, this is the first reported case of prenatal presentation of this condition with a detailed phenotypic description of the affected fetus.

## 1. Introduction

CDK13-related disorder is a newly described autosomal dominant genetic condition, caused by pathogenic variants in the *CDK13* gene. Mutations in the *CDK13* gene were first linked to human diseases by the Deciphering Developmental Disorders study in 2011 [1]. Subsequently, Sifrim et al. [2] described seven patients with CDK13-related disorders through whole exome sequencing of a cohort of probands with syndromic or nonsyndromic congenital heart defects. Currently, more than 180 patients with CDK13-related disorder have been reported worldwide (https://www.cdk13.co.uk/) with up to 62 patients being described in the literature [3]. Penetrance appears to be complete [4] and all currently reported cases are caused by *de novo* variants.

Nearly all CDK13-related disorder cases show global delay/intellectual disability, with many also experiencing feeding difficulties, hypotonia, and speech/behavioral problems. Numerous structural brain anomalies have been reported in patients including microcephaly, abnormalities of corpus callosum, periventricular leukomalacia, and cerebellar tonsillar abnormalities [5]. Common craniofacial features include hypertelorism, epicanthal folds, highly arched eyebrows, wide nasal bridge, short columella, thin upper lip, and low set/posteriorly rotated ears [6].

Cardiac anomalies were initially described as a common finding in patients with *CDK13* mutations. However, a case series by Uehara et al. [7] concluded that congenital cardiac defects are not an essential feature. In a recent review [8], just under half of reported cases had at least one cardiac malformation. Structural heart defects include ASD or VSD, abnormality of pulmonary valve or arteries, tetralogy of Fallot, bicuspid aortic valve with aortic stenosis, and LV noncompaction.

Herein, we report a prenatal presentation of CDK13-related disorder, secondary to abnormal ultrasound finding of cystic hygroma and thickened nuchal fold in the second trimester. We describe the autopsy findings and the genotype. Written consent was provided by the family for publication of clinical data.

## 2. Case Report

A 31-year-old G2P0 woman with an unremarkable prenatal history was referred to genetics after ultrasound identification of septate cystic hygroma at 17 weeks + 5 days gestational age (GA). Family history was unremarkable for congenital abnormality, recurrent miscarriages, and learning difficulties on either side of the family. Couple were nonconsanguineous and of European descent.

Previous pregnancy was an elective early termination. She was on citalopram for depression during the pregnancy. Her integrated first trimester screening showed a risk of 1 : 169 for trisomy 21 and 1 : 4710 for trisomy 18. Noninvasive prenatal screening (NIPS) showed low risk for aneuploidy in chromosomes 13, 18, and 21 and sex chromosomes. Repeat ultrasound at 20 weeks + 3 days showed nuchal fold of 8.1 mm and cystic fluid collection of 11 × 5 mm in the posterior neck. Extended cardiac views in subsequent ultrasound were normal.

Amniocentesis was performed at 17 weeks and RAD (rapid aneuploidy detection) results were normal. A next generation sequencing (NGS) in silico panel from a trio based whole exome sequencing analysis at a private laboratory was offered to the family. This panel included almost 2000 genes associated with disorders involving abnormal prenatal ultrasound findings, severe neonatal, or childhood presentation. NGS technologies were used to cover the coding regions of targeted genes plus ∼10 bases of noncoding DNA flanking each exon, with >97% of target bases at covered at 20x and mean coverage of target bases >120x. This investigation identified a likely pathogenic *de novo* variant in the *CDK13* gene, c.900C > G (p.Tyr300^*∗*^) (NM_003718.5), with no other pathogenic, likely pathogenic variants or copy number variants (CNVs) reported across the exome. Fetal echocardiogram was suggested, however parents decided to terminate the pregnancy at 24 weeks GA. Labor was induced at 24 weeks and a male fetus was delivered. The parents consented for a fetal autopsy. Growth parameters were consistent with gestational age of 24 weeks.

Autopsy report showed craniofacial abnormalities including hypertelorism, broad nasal bridge, and flattened nose, mildly prominent epicanthal folds and bilateral posteriorly rotated low set ears with residual cystic hygroma. The fetus had a prominent xyphoid process and prominent heels bilaterally. Skeletal survey was unremarkable. No cardiac anomaly was identified. A heavy spleen was reported (weight of 2 g with expected weight of 0.9 g based on GA); with a normal histopathology, the significance of this finding remains unknown. The brain and spinal cord were grossly unremarkable. No other internal structural abnormalities were noted. Neuropathology report showed multiple abnormalities including neuroblastic depletion of the germinal matrix, incompletely turned hippocampus and premature disappearance of neocortical external granule cell layer. The brainstem, spine, cerebellum, and pituitary gland were histologically normal. The variant was submitted to ClinVar (variant ID 1706567).

## 3. Discussion

In this case, ultrasound detection of cystic hygroma with nuchal fold thickening led to prenatal diagnosis of CDK13-related disorder. Fetal autopsy findings were consistent with CDK13-related disorder features previously reported in the literature. To our knowledge, this is the first reported prenatal presentation of this condition, and we propose that cystic hygroma/enlarged nuchal fold may be a prenatal feature of CDK13-related disorder.

In a retrospective study by Sukenik-Halevy et al. [9] prenatal imaging data were reviewed in 122 patients with a postnatal diagnosis of a neurocognitive disorder. In two patients diagnosed with CDK13-related disorder, there were no findings in prenatal imaging and both had a normal NT measurement. One was carrier of the common missense variant of Asn842Asp and the other had the missense likely pathogenic variant of Gly857Val.

The *CDK13* variant identified in this case (c.900C > G (p.Tyr300^*∗*^)) has not been reported in the literature to date. This variant is predicted to result in premature protein termination. Recurrent frameshift mutations previously described by Akker et al. [8] involve the kinase domain (p.Ala162fs), and these individuals were clinically indistinguishable from patients with a missense mutation.

The majority of reported *CDK13* mutations are missense with a main hotspot of Asn842Asp in almost half of the cases. Few splice site, nonsense, and frameshift mutations are currently described. More data are needed to investigate a possible phenotype-genotype relationship.

In summary, this report adds evidence that loss of function mutation in *CDK13* gene may be responsible for prenatal presentation of CDK13-related disorder with septated cystic hygroma without association with cardiac anomalies. Autopsy report in this case has shown splenomegaly with a normal histopathology, the significance of which remains unclear. Overall, large case series are required to further delineate the clinical phenotype and establish genotype-phenotype relationship.

## Data Availability

No data were used to support the findings of this study.
